# Short Hairpin RNA Library-Based Functional Screening Identified Ribosomal Protein L31 That Modulates Prostate Cancer Cell Growth via p53 Pathway

**DOI:** 10.1371/journal.pone.0108743

**Published:** 2014-10-06

**Authors:** Yojiro Maruyama, Toshiaki Miyazaki, Kazuhiro Ikeda, Toshiyuki Okumura, Wataru Sato, Kuniko Horie-Inoue, Koji Okamoto, Satoru Takeda, Satoshi Inoue

**Affiliations:** 1 Division of Gene Regulation and Signal Transduction, Research Center for Genomic Medicine, Saitama Medical University, Saitama, Japan; 2 Department of Obstetrics and Gynecology, Juntendo University School of Medicine, Tokyo, Japan; 3 Division of Cancer Differentiation, National Cancer Center Research Institute, Tokyo, Japan; 4 Departments of Geriatric Medicine and Anti-Aging Medicine, Graduate School of Medicine, The University of Tokyo, Tokyo, Japan; National Health Research Institutes, Taiwan

## Abstract

Androgen receptor is a primary transcription factor involved in the proliferation of prostate cancer cells. Thus, hormone therapy using antiandrogens, such as bicalutamide, is a first-line treatment for the disease. Although hormone therapy initially reduces the tumor burden, many patients eventually relapse, developing tumors with acquired endocrine resistance. Elucidation of the molecular mechanisms underlying endocrine resistance is therefore a fundamental issue for the understanding and development of alternative therapeutics for advanced prostate cancer. In the present study, we performed short hairpin RNA (shRNA)-mediated functional screening to identify genes involved in bicalutamide-mediated effects on LNCaP prostate cancer cells. Among such candidate genes selected by screening using volcano plot analysis, ribosomal protein L31 (RPL31) was found to be essential for cell proliferation and cell-cycle progression in bicalutamide-resistant LNCaP (BicR) cells, based on small interfering RNA (siRNA)-mediated knockdown experiments. Of note, *RPL31* mRNA is more abundantly expressed in BicR cells than in parental LNCaP cells, and clinical data from ONCOMINE and The Cancer Genome Altas showed that RPL31 is overexpressed in prostate carcinomas compared with benign prostate tissues. Intriguingly, protein levels of the tumor suppressor p53 and its targets, p21 and MDM2, were increased in LNCaP and BicR cells treated with *RPL31* siRNA. We observed decreased degradation of p53 protein after *RPL31* knockdown. Moreover, the suppression of growth and cell cycle upon *RPL31* knockdown was partially recovered with *p53* siRNA treatment. These results suggest that RPL31 is involved in bicalutamide-resistant growth of prostate cancer cells. The shRNA-mediated functional screen in this study provides new insight into the molecular mechanisms and therapeutic targets of advanced prostate cancer.

## Introduction

Prostate cancer is the fourth most common cause of cancer-related deaths, and the incidence of prostate cancer in Japan is increasing, with >11,000 deaths per year from the disease. While most early-stage, localized disease can be successfully treated by radiation therapy and/or surgery, as many as 50% of patients treated for localized disease will have local recurrence or distant metastases [1], [2]. The current first-line treatments for recurrent or metastatic prostate cancer are hormone therapies, including those that target androgen receptor (AR) signaling such as bicalutamide, and drugs such as gonadotropin-releasing hormone agonists that prevent androgen production in the testicles and adrenal glands. Although hormone therapies initially reduce the tumor burden, many patients become resistant to these therapies and develop a terminal form of the disease, termed castration-resistant prostate cancer (CRPC) [3]. Patients with CPRC have a poor prognosis and account for the majority of deaths due to the disease.

In CRPC, reactivation of AR signaling is recognized as a fundamental event that results in renewed tumor growth under conditions of androgen deprivation. Recent studies have revealed that CRPC is commonly associated with increased AR signaling due to AR amplification, AR mutation, transcription cofactor activation, ligand-independent phosphorylation of AR, and other processes [4]–[7].

Indeed, immunohistochemical studies show that overexpression of AR protein is found in most cases of CRPC [6]–[8]. These findings suggest that AR plays a central role in the development/growth of both androgen-dependent prostate cancer and CRPC [9]–[12]. AR reactivation is clinically important because AR itself and its downstream signaling pathway could be therapeutic targets in CRPC. The precise molecular mechanisms underlying AR reactivation in CRPC, however, are unclear, due to the interaction of the AR signal transduction pathway with other signaling pathways.

In the present study, we performed short hairpin RNA (shRNA) screening to identify novel genes modulating the response to the antiandrogen bicalutamide in prostate cancer cells. In a comparative study of bicalutamide-treated and vehicle-treated prostate cancer cells, volcano plot analysis [13], [14] was used to screen genes that are involved in the bicalutamide response. A cell viability assay using small interfering RNAs (siRNAs) specific for the shRNA-targeting candidate genes revealed that ribosomal protein L31 (*RPL31*), histone cluster 1 H2bd (*HIST1H2BD*), and ADAM metallopeptidase with thrombospondin type 1 motif 1 (*ADAMTS1*) were involved in the proliferation of bicalutamide-resistant prostate cancer cells. In particular, RPL31, a protein that is part of the 60S large ribosomal subunit [15]–[18], was shown to modulate the expression of the tumor suppressor p53 [19]–[21] and cell cycle regulator p21 [20]. This study reveals novel pathways modulating the bicalutamide response and therapeutic targets in prostate cancer.

## Materials and Methods

### Cell culture and antiandrogens

LNCaP, VCaP, and 22Rv-1 prostate cancer cells were purchased from the American Type Culture Collection (Manassas, VA, USA). LNCaP cells were cultured in RPMI supplemented with 10% fetal bovine serum, penicillin (100 U/mL), and streptomycin (100 mg/mL) at 37°C in a humidified atmosphere containing 5% CO_2_. VCaP and 22Rv-1 cells were cultured in DMEM with 10% fetal bovine serum, penicillin (100 U/mL), and streptomycin (100 mg/mL) at 37°C in a humidified atmosphere containing 5% CO_2_. Bicalutamide-resistant prostate cancer cells (BicR) have been described previously [22]–[24], and these cells were cultured in RPMI supplemented with 1 µM bicalutamide, 10% fetal bovine serum, penicillin (100 U/mL), and streptomycin (100 mg/mL) at 37°C in a humidified atmosphere containing 5% CO_2_. For stable transfection, LNCaP cells were transfected with C-terminally Flag-tagged RPL31 plasmid or empty vector (pcDNA3; Invitrogen) and selected in culture medium containing 0.1 mg/mL G418 (NACALAI TESQUE, Kyoto, Japan). The stable transformants of LNCaP cells expressing RPL31-Flag (LNCaP-RPL31 #39 and #63) and empty vector (LNCaP-vec #19 and #22) were cloned. Bicalutamide and docetaxel were purchased from Sigma-Aldrich Japan (Tokyo, Japan).

### Screening of bicalutamide-response-related genes using a lentiviral shRNA library

Thermo Scientific Open Biosystems Decode RNAi Viral Screening library (RHS5339) was purchased from Thermo Scientific (Huntsville, AL, USA). The shRNA screen was performed as described elsewhere [1], [13], [14], [25]. Briefly, transductions were performed in 100-mm plates such that each shRNA was represented with an average of 100 copies so that the multiplicity of infection (M.O.I.) was equal to 0.3 for median single copy integration of each shRNA. Infection of target cells at M.O.I. ≤0.3 was confirmed by fluorescence microscopy and fluorescence activated cell sorting (FACS) 48 h after infection. LNCaP cells were cultured in growth media containing 1 µM bicalutamide or vehicle (0.1% ethanol) for 1 month.

### Microarrays

Genomic DNA was isolated from transduced cells using the DNeasy Purification Kit (QIAGEN; Tokyo, Japan) according to the manufacturer's protocol. The integrated shRNAs prepared from LNCaP genomic DNAs were amplified using primers (Decode RNAi-GIPZ, annotated genes screening library-negative selection kit from Thermo Scientific) specific for the barcodes for the library plasmid DNA [13], [14], [25]. The PCR products were gel-purified using the QIAquick PCR purification Kit (QIAGEN). Purified DNA fragments (1.5 µg) from LNCaP cells treated with vehicle or bicalutamide were labeled with cyanine-3 (Cy3) or cyanine-5 (Cy5) dye, respectively, using the Genome DNA Enzymatic Labeling Kit (Agilent; Santa Clara, CA, USA) and purified by the removal of unbound cyanine dyes with an Ultracell YM-30 Microcon centrifugal filter device (Millipore Japan; Tokyo, Japan). Microarray hybridization was performed using the Oligo cDGH/ChIP-on-ChIP Hybridization Kit (Agilent). Agilent Feature Extractor software was used to scan microarray images. The GEO accession number for the microarray data is GSE60382. A volcano plot was generated by clustering based on probes. Depleted (fold change <0.5; *P*<0.01) signals in the bicalutamide-treated LNCaP cells compared with the vehicle-treated cells were selected as bicalutamide response-related genes [23], [25], [26].

### siRNA transfection and western blot analysis

Silencer select pre-designed siRNAs targeting the candidate genes were obtained from Applied Biosystems (Foster City, CA, USA) (Table 1). siRNA targeting p53 (sip53: sc-29435) was purchased from Santa Cruz Biotechnology (Santa Cruz, CA, USA). A control siRNA targeting the luciferase gene (siLuc) and a non-targeting control siRNA (siControl) with no homology to the known gene targets in mammalian cells were obtained from RNAi (Tokyo, Japan). LNCaP and BicR cells were plated in 6-well plates at a density of 100,000 cells per well and cultured overnight. Cells were transfected with siRNA at a final concentration of 10 nM using Lipofectamine RNAiMAX (Invitrogen; Carlsbad, CA, USA). Knockdown efficiency of siRNA was determined by qRT-PCR using RNA prepared from the cells 48 h after transfection and normalized with that of siControl. For western blot analysis, bicalutamide (1 µM) or vehicle was added to the medium 12 h after transfection. After 48 h, cells were harvested and lysed in a sample buffer at 100°C for 15 min for sodium dodecyl sulfate polyacrylamide gel electrophoresis (SDS-PAGE). Cell lysates were resolved on a 10% or 15% SDS-PAGE gel and then transferred to polyvinylidene difluoride membranes (Millipore Japan). Membranes were probed with one of the following primary antibodies: anti-RPL31 (Abcam; Tokyo, Japan), anti-p53 (DO-7; LeicaBiosystems; Newcastle, UK), anti-MDM2 (SMP14; Santa Cruz Biotechnology), anti-p21 (C-19; Santa Cruz Biotechnology), and anti-Flag (M2; Sigma-Aldrich). Membranes were then incubated with horseradish peroxidase-conjugated anti-rabbit IgG (GE Healthcare; Buckinghamshire, UK) or anti-mouse IgG, and visualized using enhanced chemiluminescence (GE Healthcare). Membranes were stripped and reprobed with a mouse anti-β-actin antibody (AC-74; Sigma-Aldrich) as a loading control [27].

**Table 1:** The list of genes targeted by shRNAs exhibiting bicalutamide-mediated downregulation in lentiviral library-transduced LNCaP cells and the knockdown efficiency of each siRNA chosen for validation.

Targeting gene	Fold change (bicalutamide-treated vs vehicle-treated cells)	*P* value	siRNAa)	Knockdown efficiency of siRNAb)
RPL31	0.13	6.60E-03	s12218	0.06
SFI1	0.17	2.71E-04	s18973	0.63
WWC3	0.18	6.05E-03	s31636	0.25
HIST1H2BD	0.18	7.07E-03	s6417	0.54
TMEM158	0.18	4.27E-03	s24725	0.28
SIX3	0.19	8.81E-03	not available	
PBOV1	0.20	1.56E-03	s34028	6.38
MTMR3	0.20	2.11E-03	s17013	0.34
FAHD2A	0.21	8.91E-03	s229763	0.40
ADAMTS1	0.21	6.84E-03	s18236	0.33
CRHR1	0.21	9.20E-03	not available	
ST8SIA5	0.22	5.21E-03	s26680	0.58
MAFB	0.23	7.25E-03	s19279	0.06
TCP11L2	0.23	8.70E-03	s48661	1.56
TFDP1	0.23	6.40E-03	s269604	0.11
RFC3	0.24	1.40E-03	s11949	0.60
STAT4	0.25	4.21E-03	not available	
CDC14B	0.28	4.24E-03	s16288	0.45
ANGPT2	0.28	8.48E-03	s1359	1.73
FBXO15	0.29	3.65E-04	s47366	4.17
LCT	0.30	6.91E-03	not available	
GPX7	0.31	4.12E-03	s6116	1.45
LEF1	0.32	7.24E-03	s27617	0.29
FCGR3A	0.34	1.35E-03	not available	
APOH	0.48	4.31E-03	not available	

a) Silencer select pre-designed siRNAs targeting the candidate genes were obtained from Ambion.

b) Knockdown efficiency of siRNA was determined by qRT-PCR and normalized to that of siControl.

### Quantitative PCR analysis

LNCaP and BicR cells were transfected with siRNA (10 nM) for 48 h. Total RNA was extracted from the cells using Isogen reagent (Nippon Gene; Tokyo, Japan). First-strand cDNA was synthesized from 2 µg of total RNA using SuperScript III reverse transcriptase (Invitrogen) with oligo(dT)20 primer. qRT-PCR was performed on a StepOnePlus instrument (Life Technologies) using the FAST SYBR Green Master Mix (Life Technologies) and 150 nM of each gene-specific forward and reverse primer (Table S1). The cycling conditions were 95°C for 2 min, followed by 40 cycles at 95°C for 2 s and at 60°C for 30 s. The relative differences in PCR product amounts were determined by the comparative cycle threshold method, using glyceraldehyde-3-phosphate dehydrogenase (*GAPDH*) as an internal control [27], [28]. The experiments were performed in triplicate. The Student's *t*-test was used for statistical analysis, and a probability value of *P*<0.05 was regarded as statistically significant.

### Cell proliferation assay

Cell proliferation was assessed using a kit containing WST-8 ((2-(2-methoxy-4-nitrophenyl)-3-(4-nitrophenyl)-5-(2,4-disulfophenyl)-2H tetrazolium, monosodium salt; Nacalai Tesque; Kyoto, Japan). BicR, LNCaP, VCaP, and 22Rv-1 cells were seeded in 96-well plates at densities of 2000, 4000, 16,000, and 4000 cells per well, respectively, in culture medium, and transfected with siRNA targeting either a candidate gene or luciferase (siLuc) (10 nM each) using Lipofectamine RNAiMAX on day 0. At 12 h post-transfection, cells were transferred into culture medium containing 1 µM bicalutamide or vehicle. At the indicated time points after transfection, 10 µL of a reagent solution containing WST-8 was added to each well, and the cells were incubated for 2 h at 37°C. The absorbance values for each well were measured at 450 nm on a MULTISKAN FC ELISA reader (Thermo Scientific; Ulm, Germany) [23], [28]. Representative results from >3 independent experiments are shown as mean ± s.d. of triplicated wells. Student's *t*-tests were used for statistical analysis, and *P*<0.05 was regarded as statistically significant.

### Cell-cycle analysis

LNCaP and BicR cells were transfected with siRNA (10 nM) for 12 h. The cells' media were changed into media containing bicalutamide (1 µM) or vehicle, and cells were cultured for an additional 36 h. Cells were washed once with PBS and fixed in 70% ethanol. They were then washed twice with PBS and treated with 0.2 µg/µL RNase A for 30 min. Finally, they were stained with 5 µg/mL propidium iodide (Sigma-Aldrich). Samples were quantified using a FACScalibur (Becton Dickinson; Cockeysville, MD, USA) based on DNA content, and results were analyzed with CellQuest software (Becton Dickinson) to determine the percentages of cells in the G0/G1, S, and G2/M phases [28], [29].

### Bioinformatics

The ONCOMINE database is a cancer microarray database and online data-mining platform aimed at facilitating discovery from genome-wide expression analyses [30]. The ONCOMINE database (https://www.oncomine.org/resource/login.html) was searched for candidate genes that are upregulated in prostate cancer versus normal prostate tissue by at least 2-fold (*P*<0.01). RNA sequencing data from The Cancer Genome Altas (TCGA) program [31], [32] were retrieved, and transcript per million (TPM) values for RPL31 were used as expression levels of the gene.

### Assessments for stabilization of protein

BicR cells were transfected with siRPL31 or siLuc (5 nM each) for 12 h, cultured for an additional 36 h in siRNA-free medium, and then treated with 50 µg/mL cycloheximide. Cells treated with cycloheximide for the indicated time points were subjected to western blot analysis using a p53 antibody [33], [34]. Membranes were stripped and reprobed with an anti-β-actin antibody as a loading control. p53 protein levels were quantified by densitometry and normalized to the levels of the corresponding β-actin.

## Results

### shRNA screen to identify modulators of the bicalutamide response

**Figure 1:**
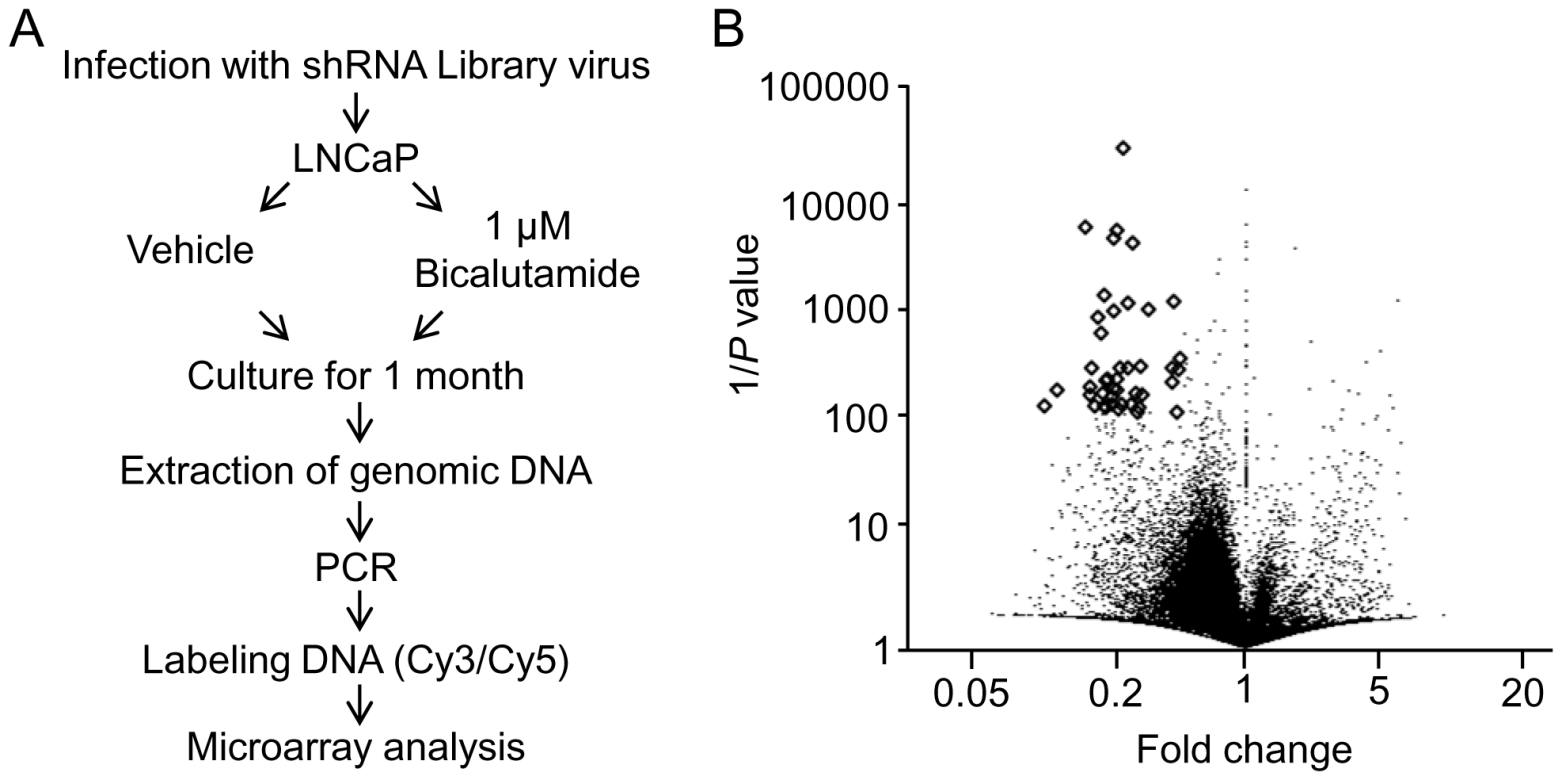
Screening for bicalutamide response-related genes in prostate cancer cells. (A) Schematic representation of shRNA screening. LNCaP cells were infected with a lentiviral shRNA library and further cultured with or without bicalutamide for 1 month. Individual integrated shRNA amounts were quantified by microarray. (B) Volcano plot of microarray data, as generated by clustering based on probes that were enriched or depleted (fold change <0.5; *P*<0.01) in bicalutamide-treated cells compared to vehicle-treated cells.

To identify candidate genes involved in the bicalutamide response in prostate cancer, we performed a functional screen by infecting LNCaP cells with a lentiviral shRNA library that comprised ∼10,000 shRNAs with unique barcodes, followed by 1 month of cell culture in the presence of 1 µM bicalutamide or vehicle (Figure 1A). In the pooled proliferation screens of cells under bicalutamide treatment, cells infected with shRNAs against genes involved in bicalutamide resistance would be removed from the cell population over time. Chromosomally integrated shRNAs were amplified by polymerase chain reaction (PCR) using genomic DNAs prepared from bicalutamide- and vehicle-treated cells, and quantified using a custom-made microarray [25], [26]. Considering shRNAs that were present multiple times or targeted to the hypothetical genes, bicalutamide treatment reduced the expression of 25 shRNAs that can target conventional genes (<0.5-fold at a threshold of *P*<0.01) compared with vehicle treatment, as shown in volcano plot analysis (Figure 1B and Table 1). This shRNA screening analysis was useful to extract genes that were putatively involved in bicalutamide resistance.

### Validation of candidate genes

**Figure 2:**
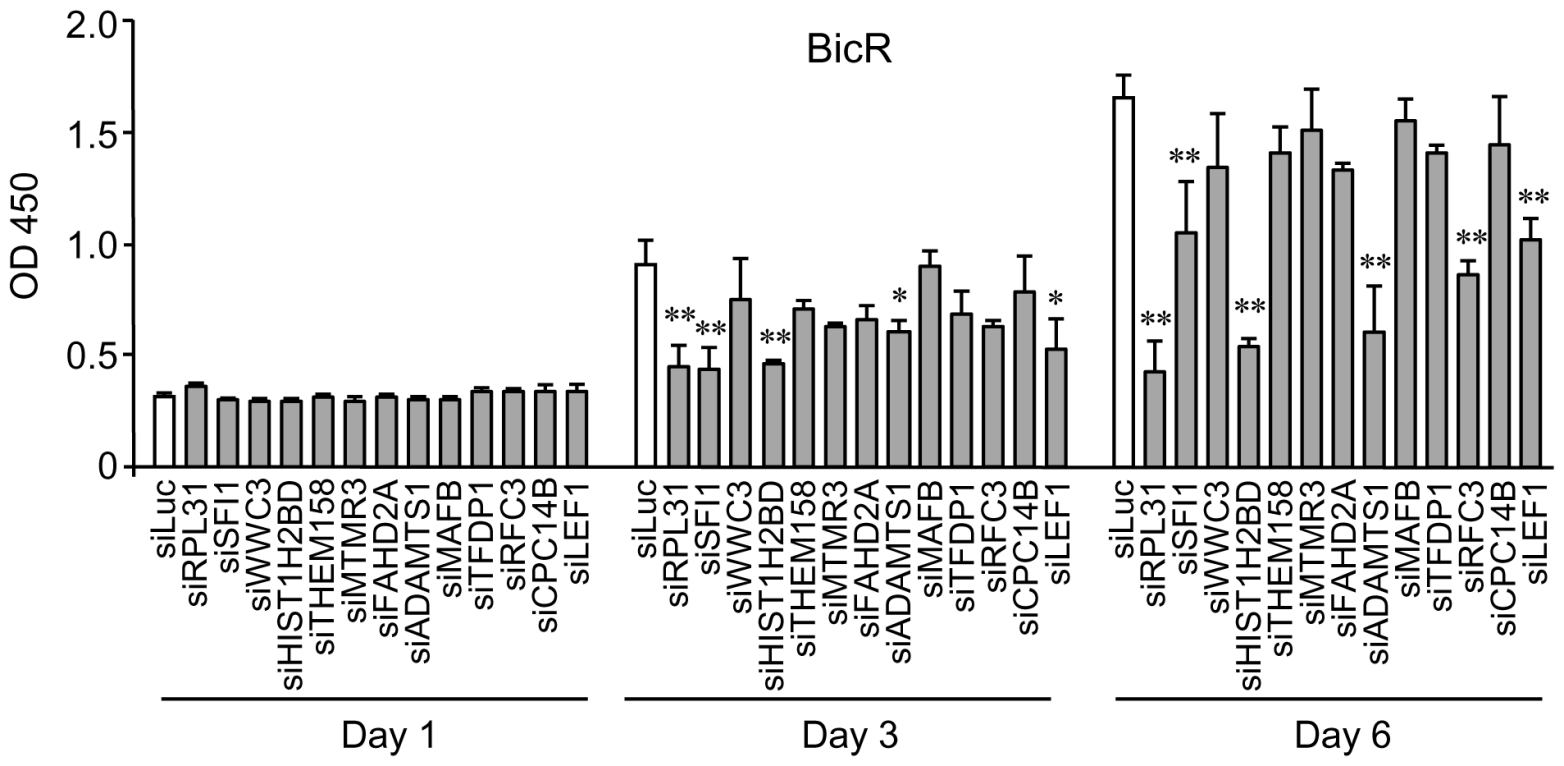
Effects on bicalutamide-resistant BicR cell growth by treatment with selected siRNAs that target genes determined by shRNA screening. Growth inhibition of BicR cells by siRNA targeting *RPL31* (siRPL31), *HIST1H2BD* (siHIST1H2BD), and *ADAMTS1* (siADAMTS1) was shown. Cells were transfected with 10 nM siRNA in culture medium. Twelve hours after transfection, cells were then further cultured in medium containing 1 µM bicalutamide. WST-8 cell proliferation assays were performed at the indicated time points after transfection. The absorbance of the wells in the plates was measured using a microplate reader at 450 nm. Data are presented as mean ± s.d. (n = 3; *, *P*<0.05; **, *P*<0.01).

To evaluate the effects of the individual genes selected by shRNA screening on prostate cancer cell biology, the knockdown efficacy of available siRNAs targeted to 19 of the 25 candidate genes was evaluated by quantitative reverse transcription (qRT)-PCR. Thirteen of the 19 siRNAs reduced the expression of their corresponding target genes by 37–94% (Table 1). Next, the effect of these siRNAs on cell proliferation in bicalutamide-resistant LNCaP (BicR) cells was assessed using the WST-8 cell proliferation assay (Figure 2). The results indicated that silencing of *RPL31, HIST1H2BD*, and *ADAMTS1*significantly repressed cell proliferation in BicR cells by >50% compared to control siRNA.

### Upregulated expression of *RPL31, HIST1H2BD*, and *ADAMTS1* in BicR cells

**Figure 3:**
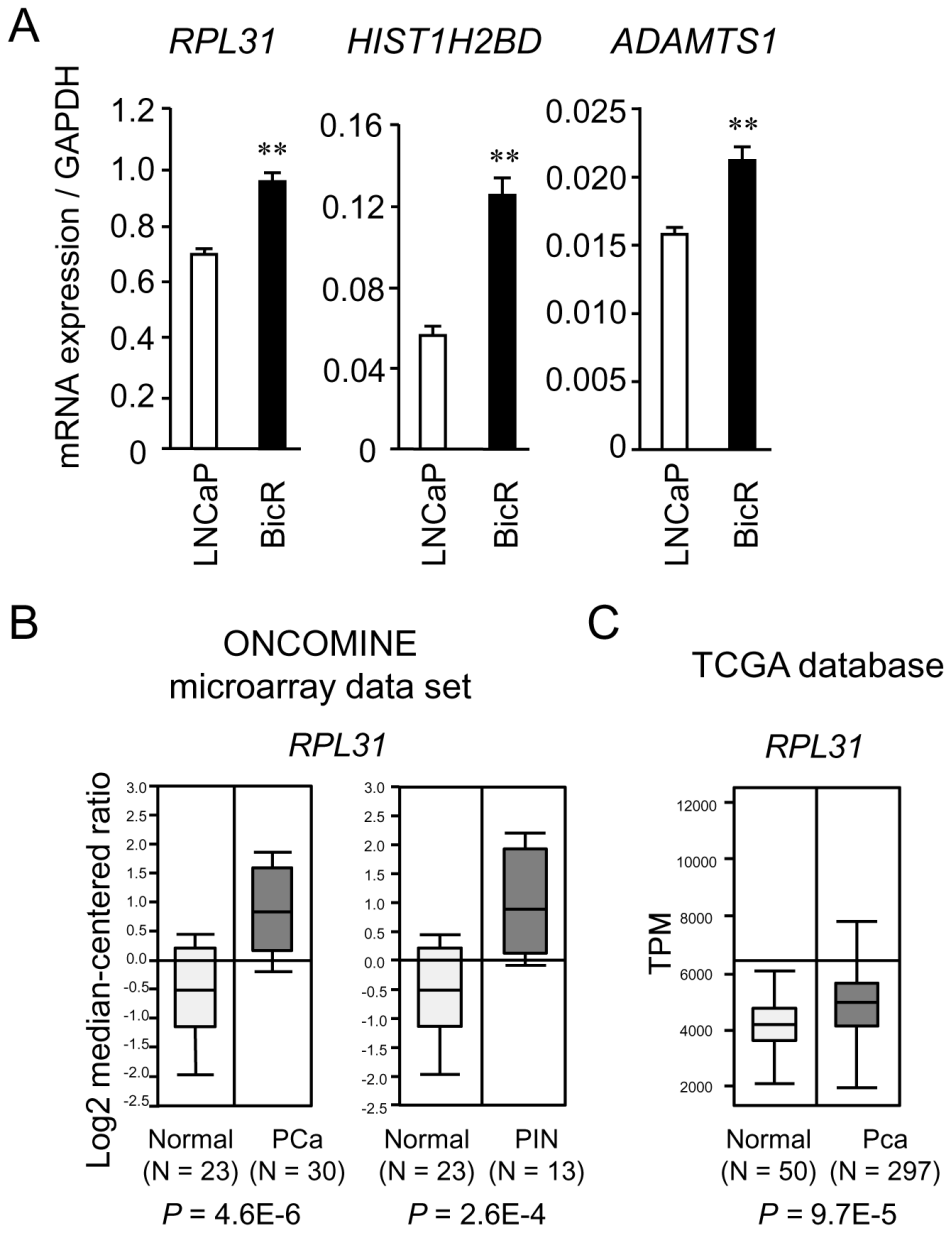
*RPL31* is overexpressed in BicR cells and clinical prostate cancer tissues. (A) Expression levels of *RPL31*, *HIST1H2BD*, and *ADAMTS1* mRNA evaluated by quantitative reverse-transcription PCR analysis (qRT-PCR) with gene-specific primers. Data are normalized to *GAPDH* and shown as mean ± s.d. (n = 3; **, *P*<0.01). (B) *RPL31* mRNA is abundantly expressed in clinical prostate carcinoma tissues compared with normal prostate tissues (by > 2-fold), as retrieved from datasets by Tomlins *et al.* in the ONCOMINE database [30]. Normal: normal prostate tissue, PCa: prostate cancer, PIN: prostatic intraepithelial neoplasia. (C) *RPL31* mRNA expression is elevated in clinical prostate cancer samples *versus* normal samples in a study of RNA-sequencing in The Cancer Genome Analysis [31], [32].

Next, we evaluated the expression levels of *RPL31, HIST1H2BD*, and *ADAMTS1* mRNA in LNCaP and BicR cells by qRT-PCR. These three genes were substantially overexpressed in BicR cells compared to parental LNCaP cells (Figure 3A). To explore whether *RPL31, HIST1H2BD*, and *ADAMTS1* expression levels were altered in clinical prostate cancer samples, we assessed the expression status of these genes based on the ONCOMINE microarray dataset [30]. In a comparison of prostate carcinoma specimens and normal prostate samples at a threshold of at least a 2-fold change (*P*<0.01) (Figure 3B), *RPL31* upregulation was observed in the study conducted by Tomlins and colleagues [35]. In an RNA-sequencing study integrated in The Cancer Genome Atlas [31], [32], *RPL31* expression was also elevated in prostate cancers compared with normal prostate tissues (Figure 3C). For *HIST1H2BD*, upregulation was shown in one dataset, whereas downregulation was shown in another (data not shown). In addition, *ADAMTS1* expression was reduced in prostate cancer in some datasets (data not shown). These results suggest that *RPL31* plays a role in prostate cancer progression, including bicalutamide resistance. To study the cell growth inhibitory effects of siRPL31 in various prostate cancer cells, VCaP, 22Rv-1, and LNCaP cells were analyzed by using a WST-8 assay. siRPL31 repressed proliferation of these cells (Figure S1).

### *RPL31* regulates cell-cycle progression

**Figure 4:**
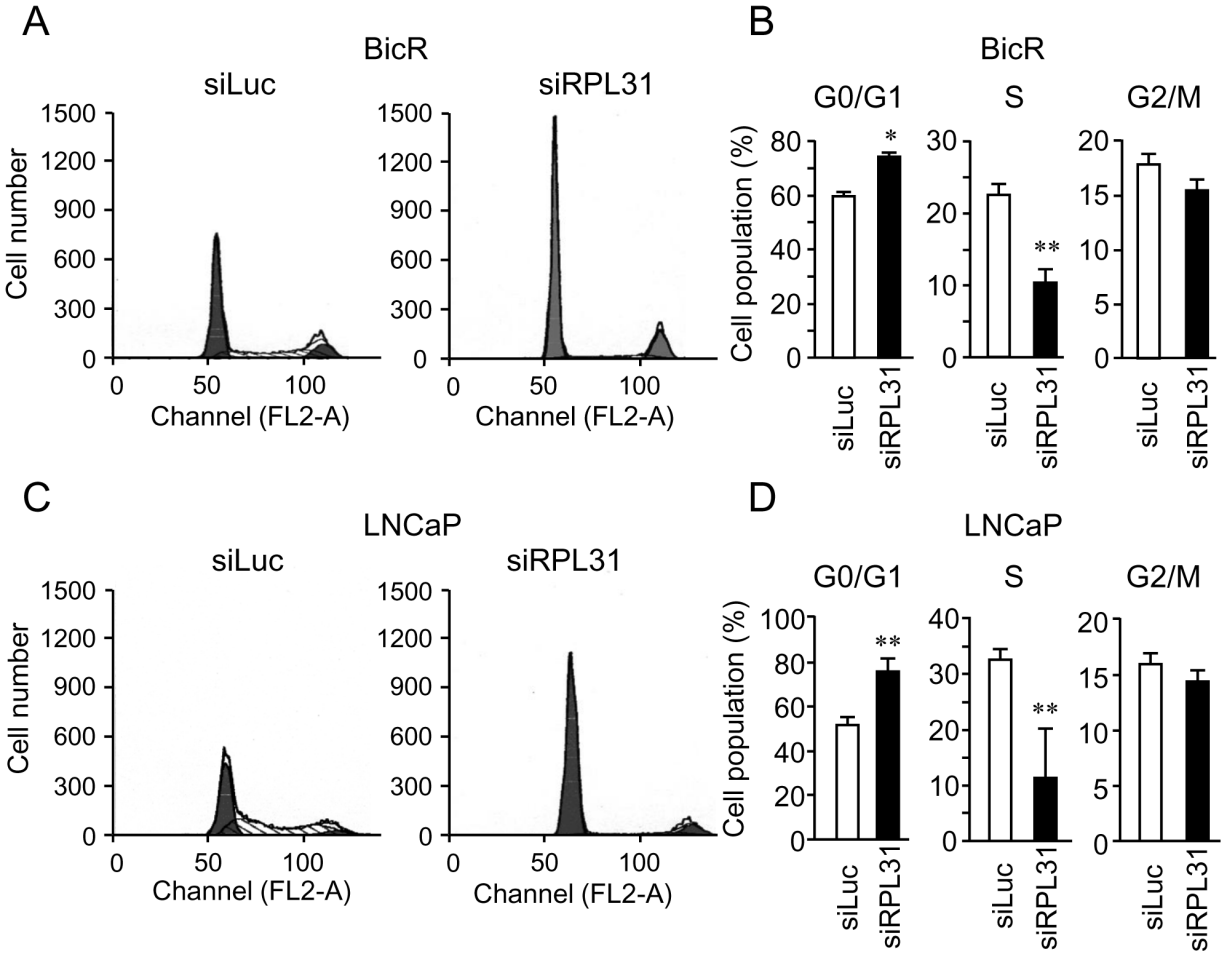
Knockdown of *RPL31* inhibits cell-cycle progression. (A) Knockdown of *RPL31* in BicR cells increased the proportion of cells in G0/G1 and decreased the proportion of those in S phase. Cells were transfected with siRPL31 or siLuc in culture medium for 48 h. Cells were then washed with PBS, stained with propidium iodide, and subjected to FACS analysis. (B) The percentages of BicR cells in S, G0/G1, and G2/M phase were determined using CellQuest software and are shown as mean ± s.d. (n = 3; *, *P*<0.05; **, *P*<0.01). (c) Knockdown of *RPL31* decreased the proliferation of LNCaP cells. Cells were treated the same as described in (A). (D) The percentages of LNCaP cells in S, G0/G1, and G2/M phases were determined using CellQuest software and are shown as mean ± s.d. (n = 3; **, *P*<0.01).

Of the siRNAs examined, si*RPL31* was the most effective at repressing BicR cell proliferation. Therefore, we further investigated the pathophysiological role of *RPL31* in prostate cancer cells. Cell cycle analysis of BicR cells revealed that *RPL31* knockdown significantly increased the proportion of cells in G0/G1 phase, while decreasing the proportion in S phase, compared with control siLuc treatment (Figure 4A and 4B). *RPL31* knockdown also induced similar cell cycle profile alterations in LNCaP cells (Figure 4C and 4D). These results indicate that *RPL31* knockdown substantially suppressed cell cycle progression of BicR cells as well as LNCaP cells.

### RPL31 modulates expression of p53 and MDM2 as well as p53 protein degradation

**Figure 5:**
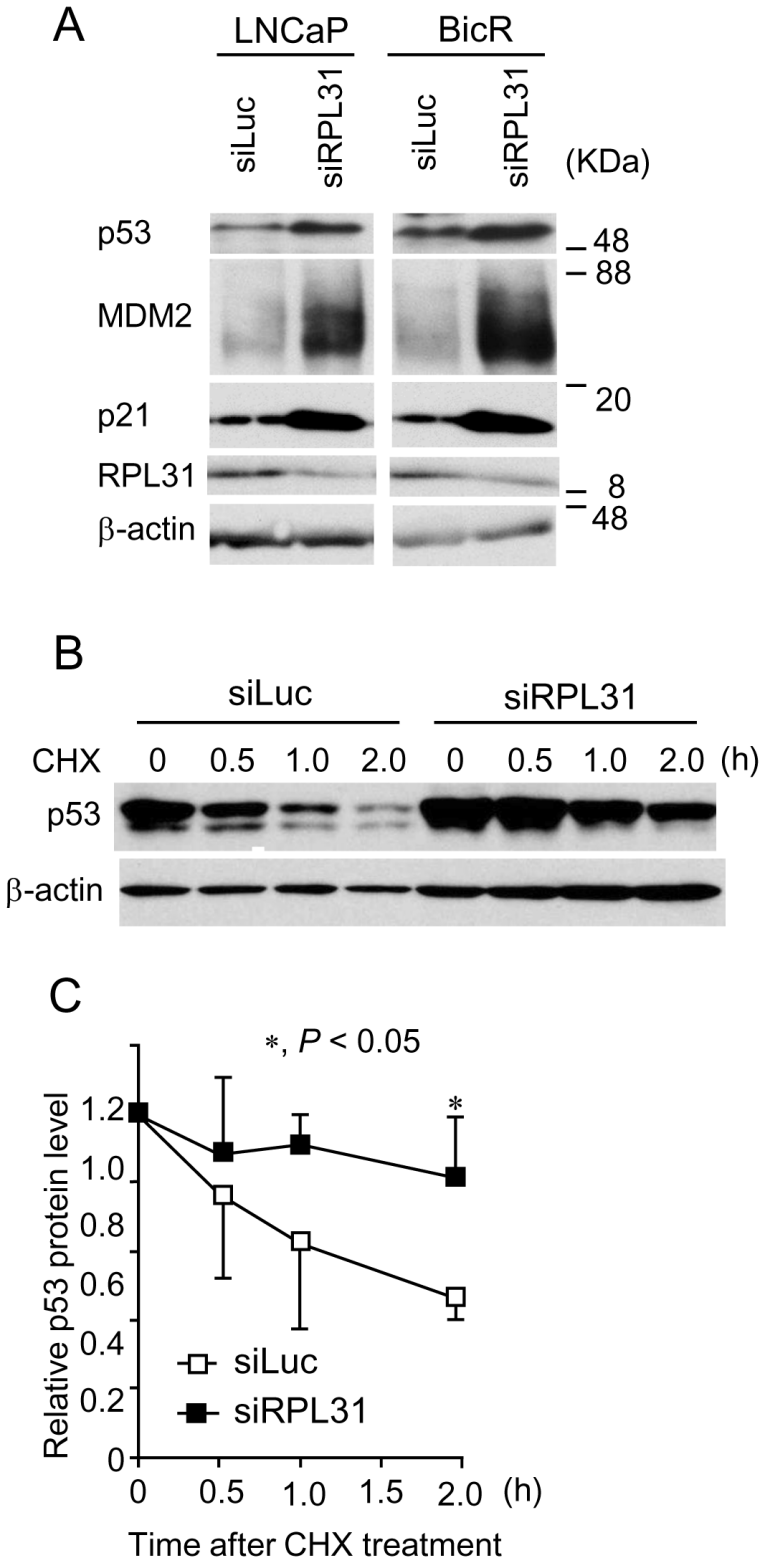
RPL31 regulates levels of p53 protein expression. (A) Knockdown of RPL31 increases p53, MDM2, and p21 protein expression. LNCaP and BicR cells were transfected with siRPL31 or siLuc for 48 h. Cell extracts were subjected to SDS-PAGE and western blot analysis using the indicated antibodies. (B) RPL31 regulated the degradation of p53 protein. BicR cells were transfected with siRPL31 or siLuc for 60 h and treated with 50 µg/mL cycloheximide (CHX) for the indicated time. Cell extracts were analyzed by western blotting. (C) p53 protein levels were quantified by densitometry and normalized to the levels of the corresponding β-actin protein and shown as mean ± s.d. (n = 3; *, *P*<0.05).

To elucidate the mechanisms underlying the role of RPL31 in cell-cycle progression of prostate cancer cells, we examined the effect of *RPL31* knockdown on the expression of cell-cycle regulators, including p53, MDM2, and p21. Intriguingly, protein levels of the tumor suppressor p53 [19], [29], [36]–[40] and its downstream cell-cycle negative regulator p21 [20] were enhanced by *RPL31* knockdown in BicR cells and LNCaP cells (Figure 5A). The expression of MDM2 protein, a known E3 ubiquitin ligase targeting p53 [21], [36], [37], was also enhanced upon *RPL31* knockdown.

We then assessed whether *RPL31* silencing influenced the degradation of p53 in prostate cancer cells. Protein levels of p53 were quantified by western blot analysis in BicR cells treated with *RPL31* siRNA and cycloheximide, an inhibitor of protein synthesis (Figure 5B). p53 protein levels were normalized to the levels of the corresponding β-actin, using densitometry (Figure 5C). p53 was stabilized more in *RPL31*-silenced BicR cells than in siLuc-treated cells.

### p53 partially mediates the cellular effects of RPL31

**Figure 6:**
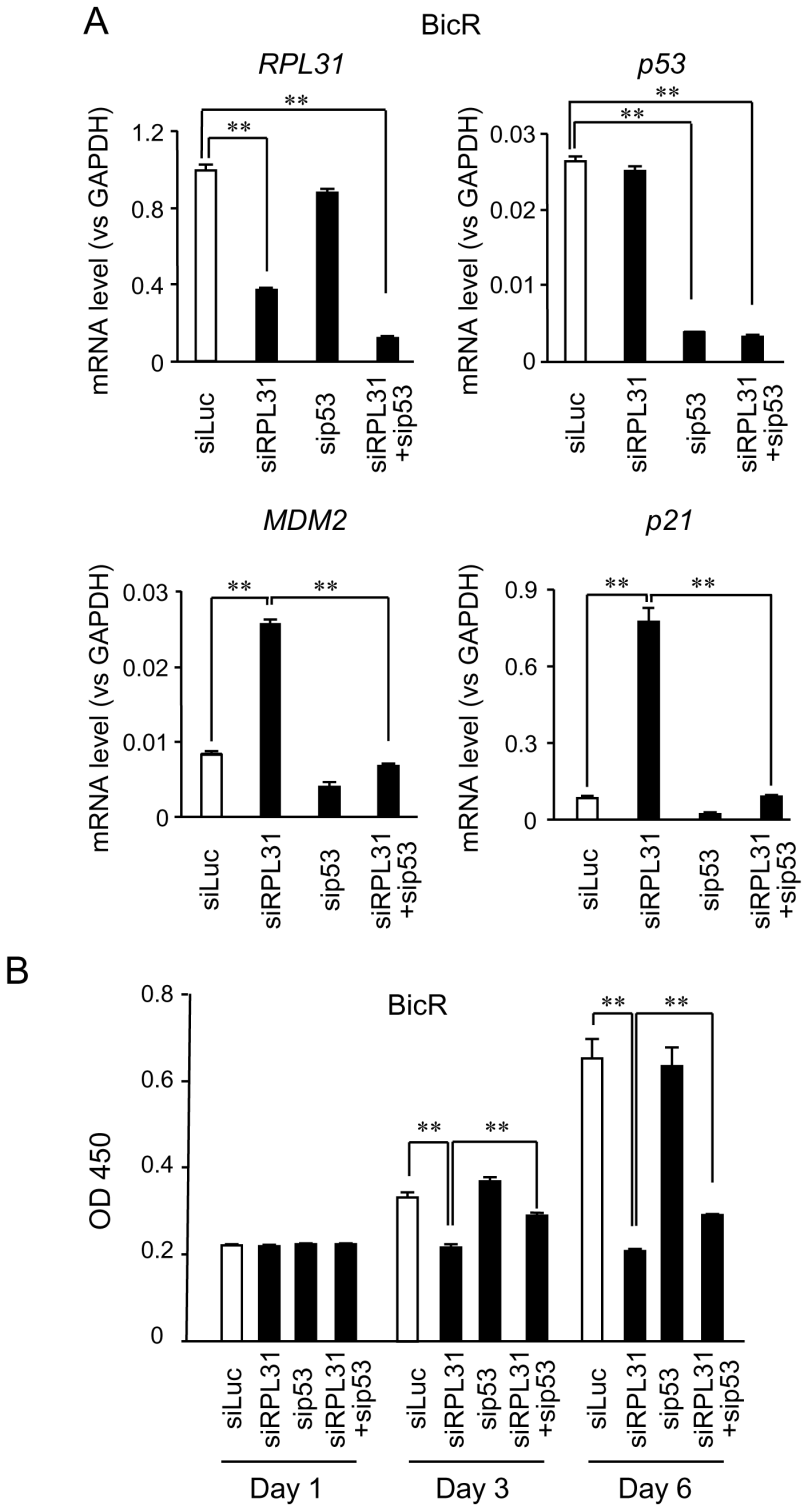
p53 partially mediates the function of RPL31 in BicR cells. (A) Knockdown effects of RPL31 and p53 on MDM2 and p21 mRNA. BicR cells were transfected with siRPL31, sip53, siRPL31 plus sip53, or siLuc. qRT-PCR for RPL31, p53, MDM2, and p21 mRNAwas performed. Experiments were performed in triplicate; mRNA expression is normalized to GAPDH and shown as mean ± s.d. (n = 3; **, *P*<0.01). (B) sip53 partially cancelled the repression of cell growth induced by siRPL31. BicR cells were transfected with 10 nM each siRPL31, sip53, siRPL31 plus sip53 or siLuc, and cultured with the medium containing 1 µM bicalutamide. WST-8 cell proliferation assay was performed at the indicated time points. The absorbances of the wells in the plates were measured using a microplate reader at a 450 nm. Data are presented as mean ± s.d. (n = 4; **, *P*<0.01).

We next examined the effects of *RPL31* knockdown on the *p53* and *MDM2* mRNA expression levels in BicR and parental LNCaP cells. *p53* mRNA levels were not increased in BicR and LNCaP cells after *RPL31* knockdown (Figure 6A, Figure S2). This finding suggests that *RPL31* silencing upregulated p53 expression at the protein level. In contrast, *MDM2* and *p21* mRNA levels were increased upon *RPL31* knockdown in BicR and LNCaP cells (Figure 6A, Figure S2), consistent with the upregulation of these proteins in both cell lines (Figure 5A).

We further verified if p53 substantially contributed to the growth inhibition mediated by *RPL31* silencing. It is notable that RPL31 knockdown-mediated upregulation of *MDM2*and *p21* mRNA in BicR cells was significantly reduced by p53 knockdown in combination with RPL31 knockdown (Figure 6A). The effects of these siRNAs on BicR cell proliferation were assessed using the WST-8 assay (Figure 6B). The results indicated that *p53* and *RPL31* knockdown partially increased cell proliferation compared with *RPL31* knockdown alone in the presence of bicalutamide. In LNCaP cells, combination of sip53 and siRPL31 also partially reversed the effects of siRPL31 on *MDM2* and *p21* mRNA expression levels as well as cell growth (Figure S2). We then examined the effect of combinatorial use of siRPL31 and sip53 on the cell cycle profile of BicR cells (Figure S3). Double knockdown of *RPL31* and *p53* increased the proportion of cells in S phase compared with *RPL31* knockdown alone.

We generated LNCaP cells expressing exogenous RPL31 by stable transfection to examine the effect of RPL31 overexpression on p53 protein (Figure S4A). It is reported that p53 protein is stabilized by a chemotherapy drug docetaxel in LNCaP cells [41]. We examined the p53 protein levels in this situation and found that the p53 protein levels were decreased in RPL31-overexpressing LNCaP cells compared with that of vector-expressing cells (Figure S4B). These gain-of-function experiments also indicated that RPL31 could negatively regulate the protein expression levels of p53. In addition, we examined whether the RPL31 expression is regulated by bicalutamide (Figure S5). Notably, it was shown that bicalutamide more strongly induced RPL31 mRNA expression in BicR cells than in LNCaP cells since, at 24 and 48 h after bicalutamide treatment, the RPL31 mRNA levels were significantly higher in BicR cells than LNCaP cells (*P*<0.01).

## Discussion

In the present study, we identified genes modulating the bicalutamide response in prostate cancer cells via functional screening using a lentiviral shRNA library combined with siRNA experiments. Out of ∼10,000 shRNAs that were transduced into LNCaP cells, we selected a small subgroup of shRNAs with substantially reduced expression in cells after 1-month bicalutamide treatment. From the 13 genes targeted by the selected shRNAs, we found that knockdown of *RPL31, ADAMTS1*, and *HIST1H2BD* significantly inhibited proliferation of BicR prostate cancer cells. We focused on the characterization of ribosomal protein RPL31 in prostate cancer biology, as silencing of *RPL31* substantially reduced cell cycle progression of BicR cells. We found that the expression level of *RPL31* mRNA was significantly elevated in BicR cells compared with LNCaP cells, and clinical studies show that RPL31 expression in prostate carcinoma is higher than that in benign prostate tissues. Taken together, these results suggest that RPL31 positively contributes to the development and phenotype of prostate cancer.

We sought to determine the mechanism by which *RPL31* knockdown severely growth arrested BicR prostate cancer cells. RPL31 is a component of the large subunit of the ribosome in eukaryotes [15], [17], [18], [42]. Because bicalutamide treatment in prostate cancer cells impairs ribosomal RNA synthesis [43], *RPL31* knockdown could further aggravate the dysregulation of ribosomal function in the presence of bicalutamide. In addition, *RPL31* knockdown could mediate extraribosomal functions and modulate the pathway of the tumor suppressor p53. Extraribosomal functions are cellular actions other than protein synthesis, including DNA replication, transcription, repair, RNA splicing, cell growth, apoptosis, differentiation, and cellular transformation [33], [34], [42], [44]–[49]. In particular, the relevance of extraribosomal functions to cell growth and cell division was confirmed by the observation that impairment of these processes upregulates p53 and causes cell-cycle arrest [36], [37]. In the present study, *RPL31* knockdown substantially increased protein levels of p53, p21, and MDM2. It is also notable that RPL31 knockdown dampened the degradation of p53 protein by cycloheximide treatment. RPL31 knockdown-mediated upregulation of *MDM2* and *p21* mRNA will be predominantly regulated by p53, as it was significantly reduced by p53 siRNA. Accordingly, RPL31 knockdown-mediated suppression of cell cycle progression and cell growth was partially rescued by p53 silencing. As speculated from the data on an *rpl31*-lacking yeast strain [17], *RPL31* knockdown would impair the assembly of ribosomal large subunits, and free ribosomal proteins will inhibit the MDM2-mediated feedback inhibition of p53 in response to the same type of stresses caused by actinomycin D [45], [46]. For example, RPS7 [44], [47], RPL5 [45], RPL11 [46], [48], and RPL23 [45], [46] upregulate p53 protein expression by binding to and inhibiting MDM2 [36], [37]. RPL5, RPL11, and RPL23 have been shown to bind to the middle region of MDM2. This region contains the acidic domain, which is critical for the MDM2-mediated p53 degradation [45], [48]. RPL11 also regulates the MDM2-p53 pathway through a post-ubiquitination mechanism [46]. Thus, it is possible that *RPL31* knockdown will lead to ribosomal protein-mediated MDM2 inhibition, p53 stabilization and activation, and MDM2 accumulation that is functionally inactive for p53 degradation. In a similar context, *RPS26* knockdown induces p53 stabilization and activation via an RPL11-dependent mechanism, resulting in p53-dependent cell growth inhibition [29]. In addition to MDM2-dependent p53 regulation, the p53-mediated upregulation of p21 also induces dephosphorylation and activation of the tumor suppressor retinoblastoma protein [50], which is a potent inhibitor of ribosomal RNA transcription and further inhibits cell cycle progression [51], [52]. We speculate that RPL31 may play an important role in the MDM2-p53 pathway cooperatively with other ribosomal subunits and ribosomal proteins. Interestingly, it was shown in the present study that bicalutamide substantially induced the expression of RPL31 in BicR cells compared with parental LNCaP cells. Taken together, these results suggest that, in response to bicalutamide, RPL31 may more strongly suppress the p53-mediateed signaling in BicR cells than in LNCaP cells. As described above, RPL31 is a ribosomal protein of the large subunit and considered to play an important role in various cellular processes. RPL31 may also basically contribute to cell viability as a ribosomal protein. Future studies are required to clarify the precise role of RPL31 in p53–MDM2 signaling.

Regarding the pathological role of RPL31 in human tumors, it has been reported that *RPL31* mRNA is upregulated in colorectal tumors [53]. In a screening of tumor antigen genes from a cDNA library, *RPL31* was identified as one of 6 genes in nasopharyngeal carcinoma [54]. Although a gain-of-function study of RPL31 isolated from the giant panda showed its anticancer function in human cancer cells [42], clinical studies reveal a tumor-promoting role for RPL31 in human malignant tissues. Of note, *RPL31* was also upregulated in clinical studies examining prostate cancer by microarray or RNA sequencing, indicating its tumor-promoting contribution in this disease as well. Interestingly, a genome-wide association study by the NCI cancer genetic markers of susceptibility (CGEMS) project and a variation of that study by a Chinese group showed that single nucleotide polymorphisms involved in the locus of circadian clock-related *NPAS2* are significantly associate with prostate cancer risk [55], [56]. *RPL31* is located adjacent to *NPAS2* in chromosome 2q11 in the same direction, and the distance between the end of *NPAS2* and the start of *RPL31* is only ∼5 kb. Thus, polymorphisms of *NPAS2-RPL31* regions are involved in a linkage disequilibrium block based on the HapMap Project data and could also affect prostate cancer risk by putatively modulating *RPL31* expression.

With regard to two other genes that were identified from shRNA screening, *ADAMTS1* is a member of the ADAMTS (a disintegrin and metalloprotease with thrombospondin motifs) metalloprotease family, which is associated with many physiological and pathological conditions. Of the 19 proteases belonging to this family, considerable attention has been paid to the role of ADAMTS1 in cancer pathophysiology [57]. It has been shown that the upregulation of *ADAMTS1* promotes pro-tumorigenic changes such as an increase in tumor cell proliferation, inhibition of apoptosis, and alteration of vascularization status [58], [59]. Our results together with these previous findings suggest that ADAMTS1 may play a role in bicalutamide resistance and other tumorigenic changes in prostate cancer cells.

*HIST1H2BD* encodes a member of the histone H2B family. Histone proteins are essential for modulating gene transcription by chromosomal modification, and the dysregulation of chromatin-associated events due to the alteration of histone modifications is thought to be responsible for cell transformation and tumorigenesis. As the dysregulation of histone H2B mono-ubiquitination has been reported to contribute to cancer development [60], HIST1H2BD may also be involved in bicalutamide resistance in prostate cancer.

In summary, we showed that functional screening of a lentiviral shRNA library and subsequent knockdown screening using siRNA was useful for identifying genes involved in the growth of bicalutamide-resistant prostate cancer cells. This approach would will provide new targets for the diagnosis and treatment of advanced prostate cancer.

## Supporting Information

Figure S1 **Silencing of** ***RPL31*** **represses the proliferation of various prostate cancer cells.** Cells were transfected with 10 nM siRPL31 and cultured. WST-8 cell proliferation assays were performed at the indicated time points. (A) siRPL31 inhibits the growth of LNCaP cells. Data are presented as mean ± s.d. (n = 4, *P*<0.01). (B) siRPL31 inhibits the growth of VCaP cells (n = 4). (C) siRPL31 inhibits the growth of 22Rv-1 cells (n = 4).
(PDF)

Figure S2 **Knockdown of** ***p53*** **partially recovers the** ***RPL31*** **silencing-mediated repression of cell growth and** ***MDM2*** **mRNA expression in LNCaP cells.** (A) Effects of *RPL31* and *p53* knockdown on the expression levels of *RPL31, p53, MDM2*, and *p21* mRNAs. Cells were transfected with siRPL31, sip53, siRPL31 plus sip53, or siLuc. *RPL31, p53, MDM2*, and *p21* mRNA levels were analyzed by qRT-PCR, performed in triplicate. mRNA expression was normalized to *GAPDH* and shown as mean ± s.d. (n = 3, *P*<0.01). (B) sip53 partially reversed the repression of cell growth induced by siRPL31. LNCaP cells were transfected with 10 nM each siRPL31, sip53, siRPL31 plus sip53, or siLuc. WST-8 cell proliferation assays were performed at the indicated time points. The absorbance values were measured using a microplate reader at 450 nm. Data are presented as mean ± s.d. (n = 4, *P*<0.01).
(PDF)

Figure S3 **Knockdown of** ***p53*** **partially impairs the inhibitory effect of siRPL31 on the cell cycle in BicR cells.** (A, B) Cells were transfected with siRPL31, sip53, siRPL31 plus sip53, or siLuc and cultured for 48 h. Cells were then washed with PBS, stained with propidium iodide, and analyzed by FACS. The percentages of BicR cells in S, G0/G1, and G2/M phases were determined using CellQuest software (panel A), and the results are shown as mean ± s.d. (panel B) (n = 3, *P*<0.05).
(PDF)

Figure S4 **RPL31 regulates p53 protein expression. (A) Generation of LNCaP cells stably expressing RPL31.** LNCaP cells were transfected with RPL31-Flag or empty vector, and stable transformants were selected with G-418. 293T cells were transiently transected with the RPL31-Flag or empty vector. Cell extracts were subjected to SDS-PAGE and western blot analysis using the Flag antibody. The stable transformants of LNCaP cells expressing RPL31-Flag (LNCaP-RPL31 #39 and #63) and empty vector (LNCaP-vec #19 and #22) were established. (B) RPL31 downregulates docetaxel induced p53 protein expression. The stable cell clones were treated with 10 nM docetaxel (Doce) or vehicle (Veh) for 48 h. Cell extracts were analyzed by western blotting using the p53 and β–actin antibodies.
(PDF)

Figure S5 **Bicalutamide upregulates the** ***RPL31*** **mRNA expression.** BicR and LNCaP cells were treated with 10–6 M bicalutamide (Bic) for the indicated times. *RPL31* mRNA levels were analyzed by qRTPCR, performed in triplicate. mRNA expression was normalized to *GAPDH* and shown as mean ± s.d. (n = 3, *P*<0.01).
(PDF)

Table S1 **The list of primers for qRT-PCR.**
(DOC)
