# Supplementary material for: Short Hairpin RNA Library-Based Functional Screening Identified Ribosomal Protein L31 That Modulates Prostate Cancer Cell Growth via p53 Pathway
Source: PLoS One. 2014 Oct 6;9(10):e108743. doi: 10.1371/journal.pone.0108743 (PMC4186824; doi:10.1371/journal.pone.0108743)
Supplement: Figure S1 — Silencing of RPL31 represses the proliferation of various prostate cancer cells. Cells were transfected with 10 nM siRPL31 and cultured. WST-8 cell proliferation assays were performed at the indicated time points. (A) siRPL31 inhibits the growth of LNCaP cells. Data are presented as mean ± s.d. (n = 4, P<0.01). (B) siRPL31 inhibits the growth of VCaP cells (n = 4). (C) siRPL31 inhibits the growth of 22Rv-1 cells (n = 4). (PDF) [file pone.0108743.s001.pdf]

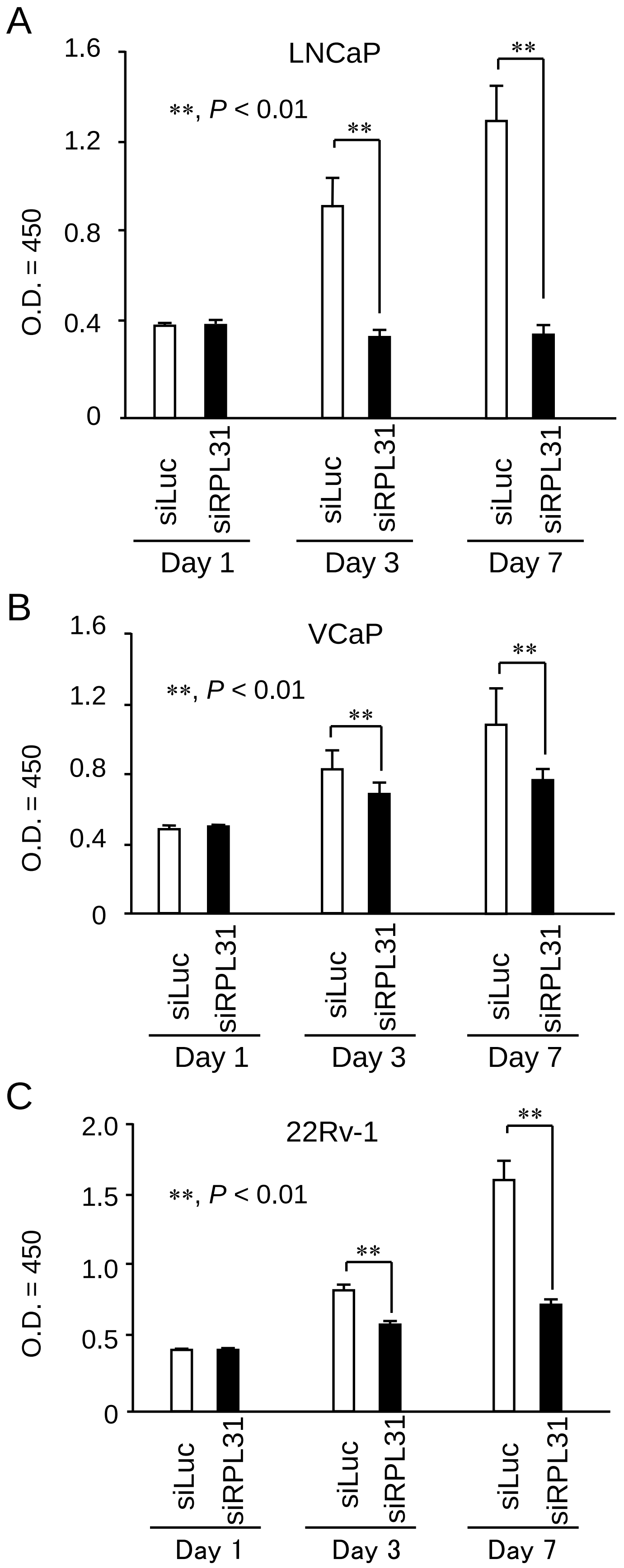

Figure S1. Silencing of *RPL31* represses the proliferation of various prostate cancer cells. Cells were transfected with 10 nM siRPL31 and cultured. WST-8 cell proliferation assays were performed at the indicated time points. (**A**) siRPL31 inhibits the growth of LNCaP cells. Data are presented as mean ± s.d. (n = 4; **, $P < 0.01$). (**B**) siRPL31 inhibits the growth of VCaP cells (n = 4). (**C**) siRPL31 inhibits the growth of 22Rv-1 cells (n = 4).
