# Supplementary material for: Short Hairpin RNA Library-Based Functional Screening Identified Ribosomal Protein L31 That Modulates Prostate Cancer Cell Growth via p53 Pathway
Source: PLoS One. 2014 Oct 6;9(10):e108743. doi: 10.1371/journal.pone.0108743 (PMC4186824; doi:10.1371/journal.pone.0108743)
Supplement: Figure S2 — Knockdown of p53 partially recovers the RPL31 silencing-mediated repression of cell growth and MDM2 mRNA expression in LNCaP cells. (A) Effects of RPL31 and p53 knockdown on the expression levels of RPL31, p53, MDM2, and p21 mRNAs. Cells were transfected with siRPL31, sip53, siRPL31 plus sip53, or siLuc. RPL31, p53, MDM2, and p21 mRNA levels were analyzed by qRT-PCR, performed in triplicate. mRNA expression was normalized to GAPDH and shown as mean ± s.d. (n = 3, P<0.01). (B) sip53 partially reversed the repression of cell growth induced by siRPL31. LNCaP cells were transfected with 10 nM each siRPL31, sip53, siRPL31 plus sip53, or siLuc. WST-8 cell proliferation assays were performed at the indicated time points. The absorbance values were measured using a microplate reader at 450 nm. Data are presented as mean ± s.d. (n = 4, P<0.01). (PDF) [file pone.0108743.s002.pdf]

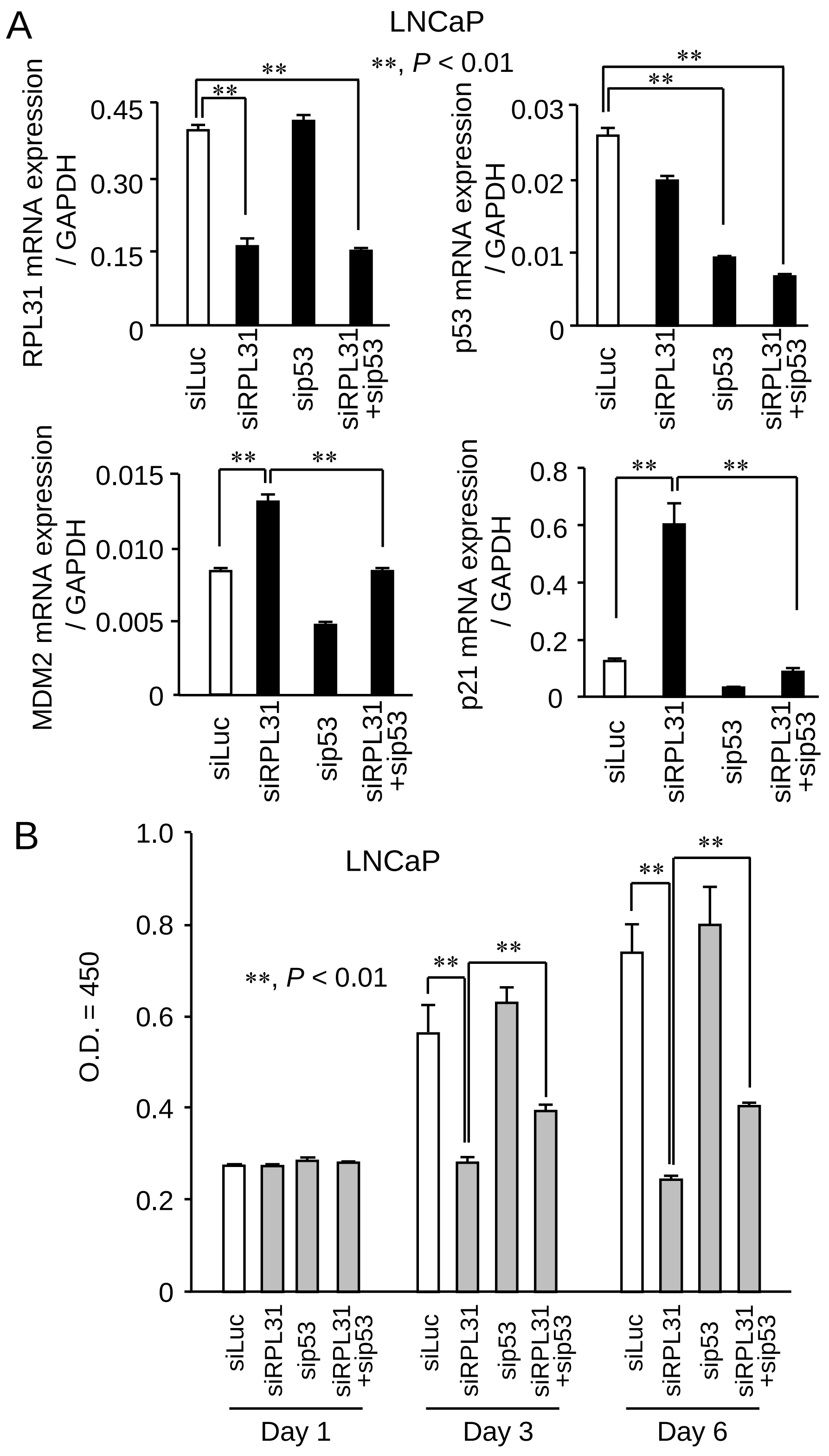

Figure S2. Knockdown of *p53* partially recovers the *RPL31* silencing-mediated repression of cell growth and *MDM2* mRNA expression in LNCaP cells. (**A**) Effects of *RPL31* and *p53* knockdown on the expression levels of *RPL31, p53, MDM2*, and *p21* mRNAs. Cells were transfected with siRPL31, sip53, siRPL31 plus sip53, or siLuc. *RPL31, p53, MDM2*, and *p21* mRNA levels were analyzed by qRT-PCR, performed in triplicate. mRNA expression was normalized to *GAPDH* and shown as mean ± s.d. (n = 3; **, $P < 0.01$). (**B**) sip53 partially reversed the repression of cell growth induced by siRPL31. LNCaP cells were transfected with 10 nM each siRPL31, sip53, siRPL31 plus sip53, or siLuc. WST-8 cell proliferation assays were performed at the indicated time points. The absorbance values were measured using a microplate reader at 450 nm. Data are presented as mean ± s.d. (n = 4; **, $P < 0.01$).
