# Supplementary material for: Short Hairpin RNA Library-Based Functional Screening Identified Ribosomal Protein L31 That Modulates Prostate Cancer Cell Growth via p53 Pathway
Source: PLoS One. 2014 Oct 6;9(10):e108743. doi: 10.1371/journal.pone.0108743 (PMC4186824; doi:10.1371/journal.pone.0108743)
Supplement: Figure S3 — Knockdown of p53 partially impairs the inhibitory effect of siRPL31 on the cell cycle in BicR cells. (A, B) Cells were transfected with siRPL31, sip53, siRPL31 plus sip53, or siLuc and cultured for 48 h. Cells were then washed with PBS, stained with propidium iodide, and analyzed by FACS. The percentages of BicR cells in S, G0/G1, and G2/M phases were determined using CellQuest software (panel A), and the results are shown as mean ± s.d. (panel B) (n = 3, P<0.05). (PDF) [file pone.0108743.s003.pdf]

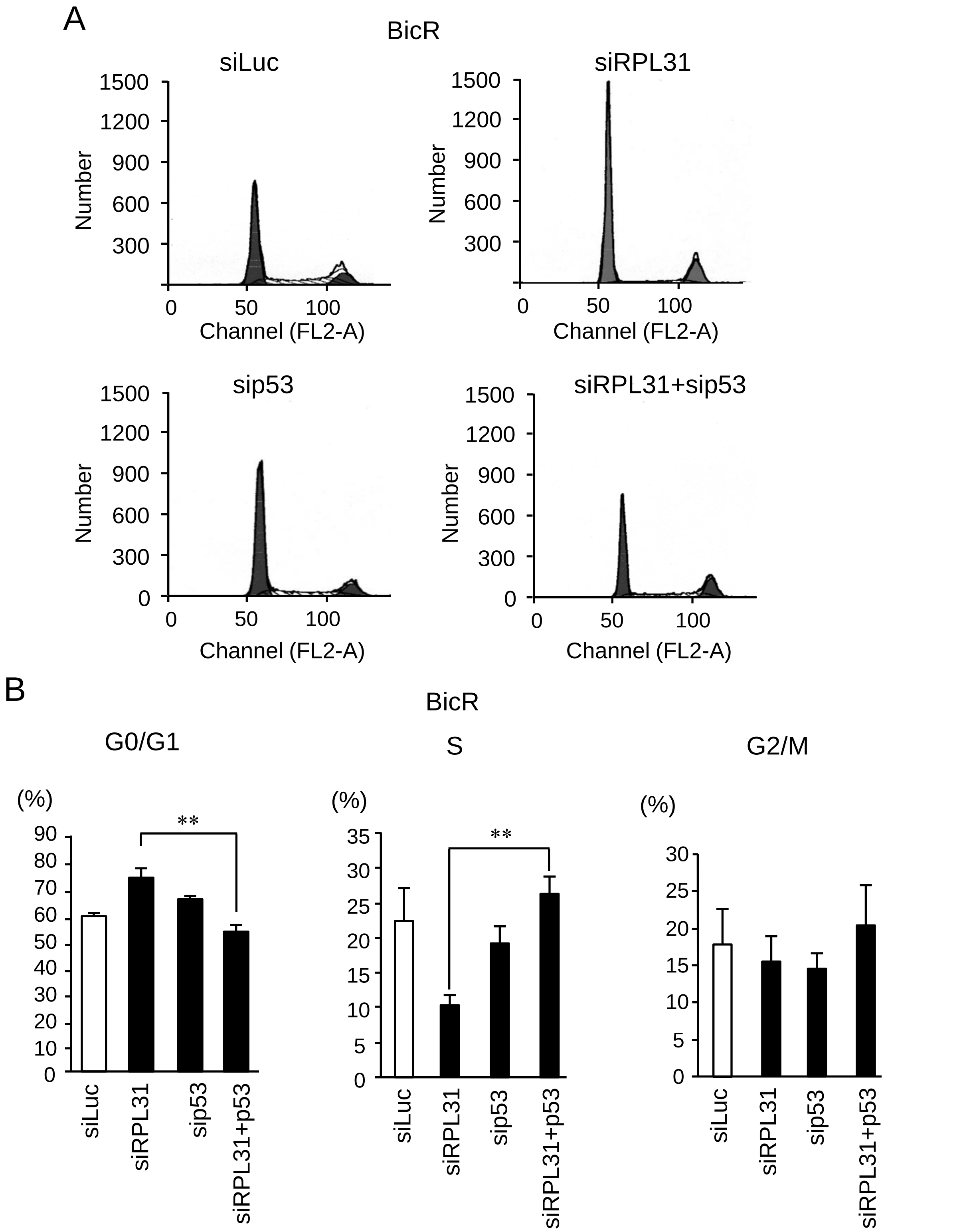

Figure S3. Knockdown of *p53* partially impairs the inhibitory effect of siRPL31 on the cell cycle in BicR cells. (**A, B**) Cells were transfected with siRPL31, sip53, siRPL31 plus sip53, or siLuc and cultured for 48 h. Cells were then washed with PBS, stained with propidium iodide, and analyzed by FACS. The percentages of BicR cells in S, G0/G1, and G2/M phases were determined using CellQuest software (panel **A**), and the results are shown as mean ± s.d. (panel **B**) (n = 3; ∗, $P < 0.05$).
