# Supplementary material for: Short Hairpin RNA Library-Based Functional Screening Identified Ribosomal Protein L31 That Modulates Prostate Cancer Cell Growth via p53 Pathway
Source: PLoS One. 2014 Oct 6;9(10):e108743. doi: 10.1371/journal.pone.0108743 (PMC4186824; doi:10.1371/journal.pone.0108743)
Supplement: Figure S4 — RPL31 regulates p53 protein expression. (A) Generation of LNCaP cells stably expressing RPL31. LNCaP cells were transfected with RPL31-Flag or empty vector, and stable transformants were selected with G-418. 293T cells were transiently transected with the RPL31-Flag or empty vector. Cell extracts were subjected to SDS-PAGE and western blot analysis using the Flag antibody. The stable transformants of LNCaP cells expressing RPL31-Flag (LNCaP-RPL31 #39 and #63) and empty vector (LNCaP-vec #19 and #22) were established. (B) RPL31 downregulates docetaxel induced p53 protein expression. The stable cell clones were treated with 10 nM docetaxel (Doce) or vehicle (Veh) for 48 h. Cell extracts were analyzed by western blotting using the p53 and β–actin antibodies. (PDF) [file pone.0108743.s004.pdf]

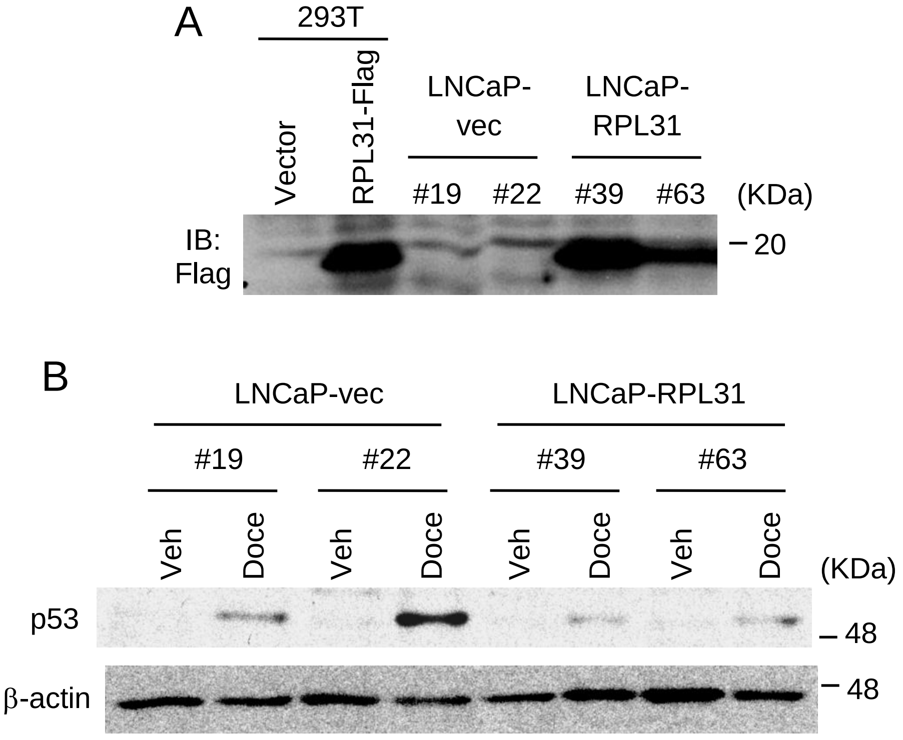

Figure S4. RPL31 regulates p53 protein expression. (A) Generation of LNCaP cells stably expressing RPL31. LNCaP cells were transfected with RPL31-Flag or empty vector, and stable transformants were selected with G-418. 293T cells were transiently transected with the RPL31-Flag or empty vector. Cell extracts were subjected to SDS-PAGE and western blot analysis using the Flag antibody. The stable transformants of LNCaP cells expressing RPL31-Flag (LNCaP-RPL31 #39 and #63) and empty vector (LNCaP-vec #19 and #22) were established. (B) RPL31 downregulates docetaxel induced p53 protein expression. The stable cell clones were treated with 10 nM docetaxel (Doce) or vehicle (Veh) for 48 h. Cell extracts were analyzed by western blotting using the p53 and β–actin antibodies.
