# Supplementary material for: Short Hairpin RNA Library-Based Functional Screening Identified Ribosomal Protein L31 That Modulates Prostate Cancer Cell Growth via p53 Pathway
Source: PLoS One. 2014 Oct 6;9(10):e108743. doi: 10.1371/journal.pone.0108743 (PMC4186824; doi:10.1371/journal.pone.0108743)
Supplement: Figure S5 — Bicalutamide upregulates the RPL31 mRNA expression. BicR and LNCaP cells were treated with 10–6 M bicalutamide (Bic) for the indicated times. RPL31 mRNA levels were analyzed by qRTPCR, performed in triplicate. mRNA expression was normalized to GAPDH and shown as mean ± s.d. (n = 3, P<0.01). (PDF) [file pone.0108743.s005.pdf]

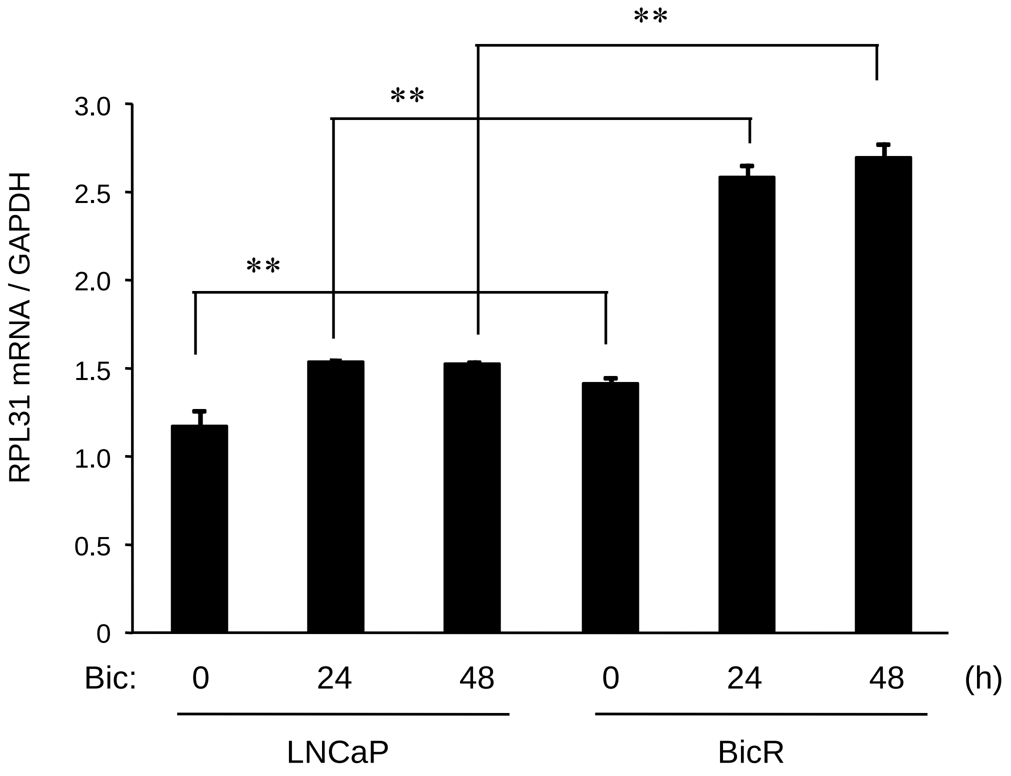

Figure S5. Bicalutamide upregulates the *RPL31* mRNA expression. BicR and LNCaP cells were treated with 10^{-6} M bicalutamide (Bic) for the indicated times. *RPL31* mRNA levels were analyzed by qRT-PCR, performed in triplicate. mRNA expression was normalized to *GAPDH* and shown as mean ± s.d. (n = 3; **, $P < 0.01$).
