# Supplementary material for: Short Hairpin RNA Library-Based Functional Screening Identified Ribosomal Protein L31 That Modulates Prostate Cancer Cell Growth via p53 Pathway
Source: PLoS One. 2014 Oct 6;9(10):e108743. doi: 10.1371/journal.pone.0108743 (PMC4186824; doi:10.1371/journal.pone.0108743)
Supplement: Table S1 — The list of primers for qRT-PCR. (DOC) [file pone.0108743.s006.doc]

| **Table S1.** The list of primers for qRT-PCR | | |
| --- | --- | --- |
| Gene symbol | Primer | |
|  | Forward | Reverse |
| RPL31 | CAGAATGGCTCCCGCAAA | TGGCAGAACGGCCCTTT |
| SFI1 | ACTGGCAAAAACGGAGATTTCTA | CTGTCTCTTCACTCTGCGGACTT |
| WWC3 | GCCTTACAGCCCCGAGAAAT | AGATCTTCCGTGTTGGTTTCCTT |
| HIST1H2BD | TCCCGACACCGGCATCT | TCGTTGACGAAGGAATTCATGA |
| TMEM158 | CCCCTGCCCGACTCCTT | ATTTGGTCATTTTCTGCCATGA |
| PBOV1 | AGGCTGAGGCGGAGAATTG | TCTCAGCTCACCGCAACCT |
| MTMR3 | GGTCTTAGCACCCTCCAGATGTA | CAGTGAGTCCTTGCTCCTACCA |
| FAHD2A | TGGTGTGTGTGGGCATGAAT | GCACGGGCACGTTCTGTT |
| ADAMTS1 | GCTCATCTGCCAAGCCAAAG | ATCTACAACCTTGGGCTGCAA |
| ST8SIA5 | TGCAGGCCCTTGTTGCA | CCTGTCTCCACTCTCAATTCCAT |
| MAFB | CCTTCCCTTATCCCCATTCG | GCACCAACAAGGTTGAAAACTAAA |
| TCP11L2 | CAAGGCCTGGCCAACTATGT | CGCACGGGAGCACACA |
| TFDP1 | GTCATCCACCTGCCCTTCA | TGCAGTCGATGACCGTCTTC |
| RFC3 | GCCGTCACCCCAAAAAGTT | GGGCCAGGGTAGGGATGA |
| CDC14B | GTATGATGCCAAACGCTTTACG | CATCCGCAAAGAAAAGATCATG |
| ANGPT2 | AGCATGGGTCCTGCAGCTA | GAGGAAGAGCGGCAGTTGTC |
| FBXO15 | GCGGCCCCGCTCTCT | GGCCTTGCGCCAAGCT |
| GPX7 | CCGCCGCACCTACAGTGT | CCAGTACCGGTGACTGCAATC |
| LEF1 | CAGGAGCCCTACCACGACAA | CCTCCATCTGGATGCTTTCC |
| p53 | CCCAGCCAAAGAAGAAACCA | CACGCCCACGGATCTGA |
| p21 | CTGGAGACTCTCAGGGTCGAA | CGGCGTTTGGAGTGGTAGAA |
| MDM2 | CGACCTAAAAATGGTTGCATTG | GCACATGTAAAGCAGGCCATAA |
| GAPDH | GGTGGTCTCCTCTGACTTCAACA | GTGGTCGTTGAGGGCAATG |
